# Recent Advancements in Metallic Drug-Eluting Implants

**DOI:** 10.3390/pharmaceutics15010223

**Published:** 2023-01-09

**Authors:** Sadeq Alshimaysawee, Rasha Fadhel Obaid, Moaed E. Al-Gazally, Andrés Alexis Ramírez-Coronel, Masoud Soroush Bathaei

**Affiliations:** 1 Faculty of Pharmacy, Islamic University, Najaf, Iraq; 2 Department of Biomedical Engineering, Al-Mustaqbal University College, Babylon, Iraq; 3 College of Medicine, University of Al-Ameed, Karbala, Iraq; 4 Laboratory of Psychometrics, Comparative Psychology and Ethology (LABPPCE), Universidad Católica de Cuenca, Ecuador and Universidad CES, Medellín, Colombia, Cuenca, Ecuador; 5 Department of Materials Engineering, Science and Research Branch, Islamic Azad University, Tehran, Iran

**Keywords:** implants, localized drug delivery, bioactive coatings, infection, biomaterials, bone tissue engineering

## Abstract

Over the past decade, metallic drug-eluting implants have gained significance in orthopedic and dental applications for controlled drug release, specifically for preventing infection associated with implants. Recent studies showed that metallic implants loaded with drugs were substituted for conventional bare metal implants to achieve sustained and controlled drug release, resulting in a desired local therapeutic concentration. A number of secondary features can be provided by the incorporated active molecules, including the promotion of osteoconduction and angiogenesis, the inhibition of bacterial invasion, and the modulation of host body reaction. This paper reviews recent trends in the development of the metallic drug-eluting implants with various drug delivery systems in the past three years. There are various types of drug-eluting implants that have been developed to meet this purpose, depending on the drug or agents that have been loaded on them. These include anti-inflammatory drugs, antibiotics agents, growth factors, and anti-resorptive drugs.

## 1. Introduction

**Figure 1:**
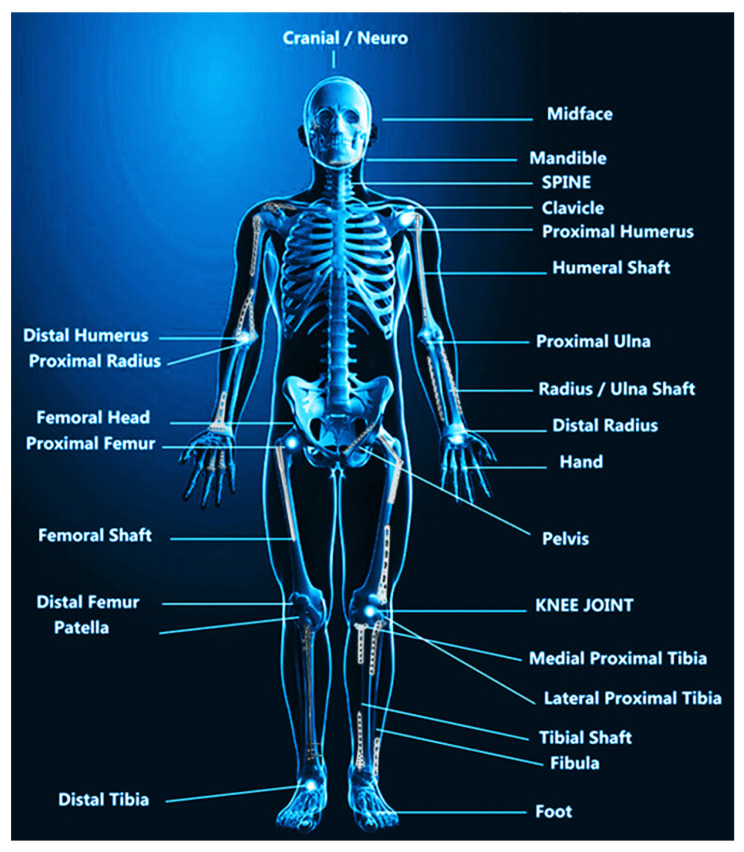
The clinical application of common metal implants. Reprinted with permission from the reference [9].

The volume of orthopedic surgeries is quickly increasing due to the aging population and osteoporosis’ significant increase, so the development of novel orthopedic implants is crucial [1,2]. Orthopedic implants accounted for $46.7 billion in the U.S. market in 2018, and are expected to grow to $66.0 billion by 2026 [3]. Joint reconstruction represents the largest share (41.2%) of the market, followed by spinal, trauma, orthobio-logics, and dental implants [4]. Approximately 22% and 16% of implant-related failures are caused by stress-shielding (i.e., Wolff’s principle) and infection, respectively [3,4]. A majority of orthopedic implants are made of metals and their alloys, such as titanium (Ti), tantalum (Ta), magnesium (Mg), zinc (Zn), stainless steels, and cobalt (Co)-based alloys, due to their low-cost and stability [5,6]. They offer an excellent combination of plasticity and toughness, along with favorable mechanical properties, that make them highly efficient [7]. There are two types of implants: temporary fixation devices, such as bone plates, pins, and screws, and permanent implants, such as total joint replacements in orthopedics [8]. There is the clinical application of common metal implants as shown in Figure 1 [9].

Implant stabilization and long-term success, largely depend on the quality of integration with the surrounding tissue [10]. The implant material, the quality and quantity of formed surrounding bone tissue and the presence of microbial infection all play a crucial role in the integration of the surrounding tissue with the implant [11]. “Stress shielding” effect can be mentioned among other factors that are responsible for implant loosening. During the “stress shielding” effect, the mismatch in modulus between the bone and implant interface results in decreased physiologic loading of the bone when the metal implants are fixed at the fractured site [11,12]. In spite of the fact that metallic implants have good mechanical properties and are generally affordable, their insufficient biological activity poses a disadvantage [12,13]. The low corrosion resistance, tendency to infection, lack of proper biological activities, and subsequent weak integration with contacted bone tissue are some of the primary concerns that drive to develop multifunctional and bioactive metallic implants that act as local drug delivery platforms [13,14,15]. In order to influence the regeneration process dynamically, they are first supposed to interfere with the response of the host body, then increase the integration with the implant, promote osteoconduction and the angiogenesis on the surface of them, and finally slow down the microbial infection process. All these steps will lead to increased tissue healing speed [16,17,18]. It is particularly promising to use localized therapeutic strategies because they have a better bioavailability, and they result in immediate bone healing as opposed to systemic therapies [19,20,21]. An effective way to enhance bone healing and regeneration is to administer biologically active compounds that induce messages that influence bone healing in a controlled manner [22,23]. Moreover, by increasing the drugs’ dose near the implant, they can avoid common systematic toxicity associated with traditional drug administration methods [24,25]. Different types of locally delivered molecules can be used to treat musculoskeletal syndromes, including nonviral genes (DNAs, RNAs), antibiotics, anti-inflammatory ingredients, proteins, growth factors, and enzymes [26].

One of the most notable applications of drug-eluting implants in bone tissue engineering is the prevention of associated infections with dental implants and orthopedic implants [27,28,29,30]. The majority of metal-based drug delivery involves embedding drugs into polymeric or ceramic coatings applied to metallic implants [31]. There are also methods of incorporating the drug itself onto the metallic implant surface using covalent bonds, self-assembled layers, and silver nanoparticles [32,33]. Meanwhile, deposition of polymer-based layers are believed to cause complications, such as loosening from the implantation site, changes in chemical composition in physicochemical media, and likely side effects due to the corrosion products [34,35]. For this reason, many researchers have been investigating the use of inorganic coatings as drug delivery systems [36]. There has been little attention given to metallic drug eluting systems in comparison with polymeric systems. This mini-review aims to summarize recent advancement in drug delivery systems on the surface of metallic implants, mainly for orthopedic and dental applications. In this review, we do not intend to provide an exhaustive synopsis of the field of drug delivery—which is vast—but highlight curiosities and advances between 2019–2022 about drug delivery systems on metallic implants. In the mentioned time period, various therapeutics substances, such as anti-inflammatory drugs, antibiotics agents, growth factors, and anti-resorptive drugs, have been loaded and eluted from metallic implants. It should be noted that most of the studies in this period concerned the development of drug-eluting implants based on Ti as a substrate, loaded with gentamicin as a therapeutic agent. Moreover, some of the studies have been focused on development of smart coatings as drug delivery platform on metallic implants.

## 2. Conventional Drug-Eluting Implants

### 2.1. Anti-Inflammatory Drug-Eluting Implants

Foreign bodies such as implantable medical materials commonly trigger immune reactions and inflammatory cascades [37,38]. There is a wide range of reactions that can affect the patient’s quality of life and the effectiveness of the implanted material, ranging from pain to swelling to rejection [39]. Anti-inflammatory and immunosuppressive drugs can be delivered in various ways to counter inflammation, which is a vital factor affecting regeneration [40]. The matrix or surface of metallic implants has been profitably used to deliver multifunction drug and anti-inflammatory drugs, such as betamethasone and dexamethasone, to reduce the kinetics of foreign body reactions around the implantation site and the production of fibrous capsules [41,42]. In nanomedicine, recent advances have made it possible to deliver drugs over time while maintaining their bioactivity [43]. It has been reported that 3 weeks after implanting silicon and platinum-polyimide neural probes with dexamethasone-loaded nanoparticles on the implant surface, tissues are significantly less prone to react with them [44,45]. It is worth mentioning that cytokine delivery has also been proven to be an efficient method of modulating the immune response to implants, since they play an essential role in regulating immune cell phenotypic changes. [45,46,47].

**Figure 2:**
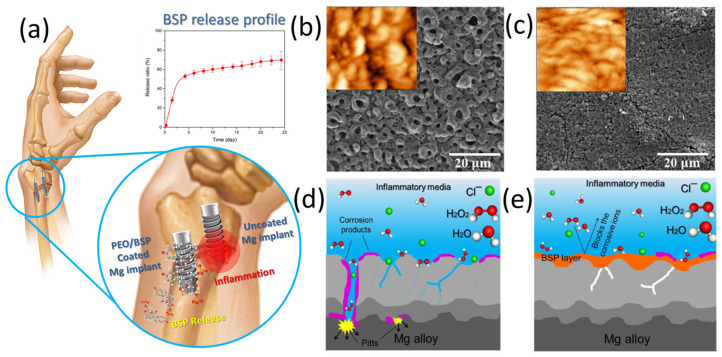
(**a**) The schematic illustration and release profile of PEO/BSP coating on Mg alloy; Scanning electron and atomic force micrographs of (**b**) PEO, and (**c**) PEO/BSP coatings; The corrosion performance of Mg implants with different coatings in simulated inflammatory condition: (**d**) PEO, and (**e**) PEO/BSP coatings. Reprinted with permission from the reference [53].

It is possible to create a smart biomaterial by simply immersing porous ceramic coated implants in pharmaceutical solutions and growth factors that penetrate directly into coating pores [48]. Initially, drugs were carried by stand-alone calcium phosphate (Ca-P) compounds deposited onto metal substrates [49,50]. This review does not cover these topics and they can be found elsewhere [51]. In recent years, plasma electrolytic oxidation (PEO) method has been studied for improving metallic implants’ corrosion resistance [52]. In this method, the micro-porous oxide layers are grown on a metallic substrate during oxidation process [52]. The porous oxide layer can act as a polymer-free drug delivery platform [53]. It was recently reported that an anti-inflammatory betamethasone sodium phosphate (BSP) drug was loaded into a PEO layer of Mg alloy [53]. It has been shown in this study that using a combination of PEO/BSP coating over a simple PEO coating provides more surface protection to Mg alloy in simulated inflammatory condition than a simple PEO coating alone (Figure 2).

Up to now, nanostructured drug loaded surfaces have been demonstrated to have anti-inflammatory, cytokine producing, and macrophage polarizing effects. The development of nanostructured drug-eluting surfaces has been associated with the formation of polarized macrophages by modulating the shape and plasticity of macrophages, stimulated by integrin beta signaling pathways. However, it is still unclear exactly how these immune-modulating mechanisms operate at a nano-scale.

### 2.2. Antibiotics-Eluting Implants

**Figure 3:**
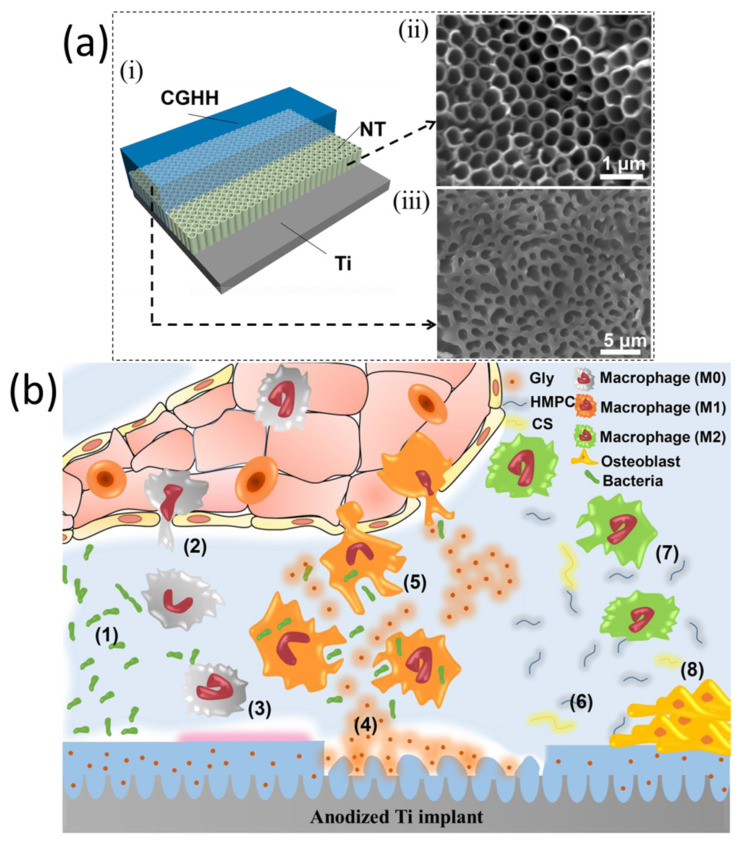
(**a**(**i**)) schematic illustration of the CGHH@NT sample; (**a**(**ii**)) SEM image of the NT sample; (**a**(**iii**)) SEM image of the CGHH@NT sample, (**b**) schematic illustration of the thermo-sensitive immunoregulation of the CGHH@NT sample: (1) bacterial infection; (2) macrophages recruitment; (3) local temperature increase; (4) phase transformation of CGHH@NT from sol state to gel state, leading to the release of Gly, (5) macrophages were polarizing toward M1 phenotype, and played their roles in bacteria killing, (6) local temperature decrease resulting in the phase of CGHH@NT reverse transforming to a sol state and releasing HPMC and CS, (7) macrophages were induced to a M2 phenotype, (8) tissue healing were promoted. Reprinted with permission from the reference [68].

It is estimated that over half of all hospital acquired infections are caused by post-surgical implant-associated infections [54]. As a result, synthetic orthopedic implants are commonly used to deliver antibiotics locally at the implantation site [55]. An infection at the site of implantation is normally caused by bacteria on the patient’s skin or in the body that have the potential to become pathogenic by adhesion and colonization from the outside (the area surrounding the implantation site, surgical instruments, etc.) [56]. Surgery and irrigation are typically performed to manage such infections, implant removal is often required, and extended antibiotic treatments are often needed [57]. This can lead to trauma to the patient, prolonged hospitalizations, and serious social and health problems [58]. Therefore, the development of implants that are intrinsically antibacterial will decrease the risks of upcoming complications and possibly reduce the large social and economic burden that may be associated with these complications [58]. Surface topography and surface chemistry have been used to achieve anti-biofouling properties by integrating antibacterial agents into implants [59]. Several implant-based strategies exist, including coatings, bone cement, composite materials, or polymethylmethacrylate (PMMA) beads loaded with antibacterial agents [60]. The success of such approaches has been largely attributed to lower infection rates. However, their initial release profiles and burst releases have not been optimized [60]. There are several downsides to the current methods, including inadequate bonding between coating and substrate. The retrieval surgery is also required to remove PMMA beads with non-biodegradability properties [61]. Among the many antibiotic agents available, choosing the appropriate agent is vital since few antibiotics have been demonstrated to adversely affect osteogenic cells at bactericidal dosages [62]. Nanotechnology has led to advances in the field of nanoscale surface modification for Ti implants for drug delivery [63]. These nanoscale modifications in the range of 1–100 nm can increase protein attachment, enhance bone-implant contact, and improve osseointegration [63]. Acid etching, electrochemical anodization, and lithography can be used to fabricate nano-topography on Ti [64]. It is important to recognize that among these strategies, electrochemical anodization has been found to be the most reliable, cost-effective, and scalable technique to fabricate nanostructures on the surface of the Ti implants, such as titanium oxide (TiO_2_) nanotubes (NT) [65]. The use of TiO_2_ NT on Ti implants is a superb surface engineering technique and drug therapies can be enabled by using such technologies, which are capable of achieving excellent results [66,67]. The glycerin (Gly)-loaded thermo-sensitive chitosan (CS)/hydroxypropyl methylcellulose (HPMC) on the anodized Ti surface has been used as coating material, called CS-Gly-HPMC hydrogel (CGHH@NT), for reducing the bacteria-associated infection after implantation (Figure 3) [68]. The finding of the study demonstrated the ability of Gly to inhibit the inflammatory response, induce macrophages to polarize towards an anti-inflammatory M2 phenotype, and generate anti-inflammatory cytokines, which enhance tissue regeneration.

### 2.3. Growth Factor-Eluting Implants

**Figure 4:**
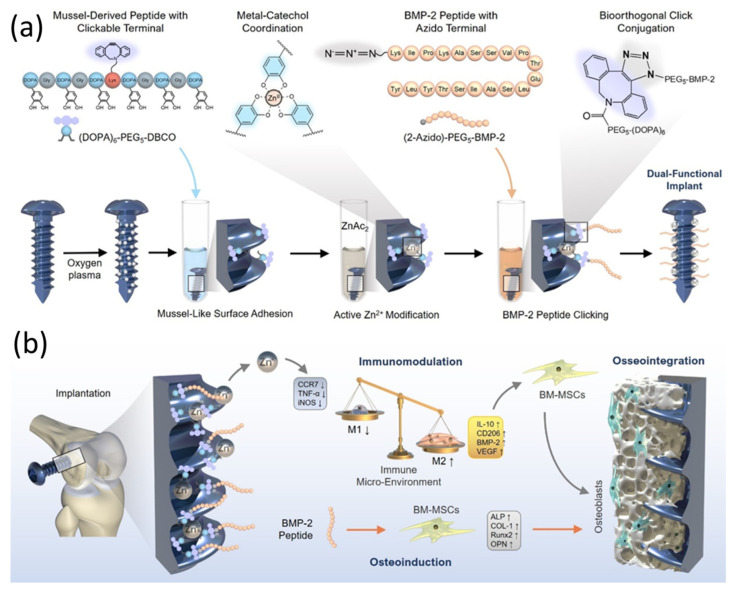
(**a**) Schematic illustration of the mussel-derived peptide for ion coordination and biomolecular click conjugation on a medical Ti screw, (**b**) In a bone implant model, the Zn^2+^ and BMP-2 peptide co-modified Ti screw shows osteoinductive and immunomodulatory dual functions in vivo, synergistically enhancing the interfacial osteogenesis and the intra-bone implant integration after implantation. Reproduce and adapted from [80] under Creative Commons Attribution 4.0 International License (CC BY 4.0).

In osteointegration, bone formation, remodeling, and impaired healing, growth factors (GFs) play an indisputable role in cell function at the local level as a large number of polypeptides [69]. By accelerating osteoclastic resorption and promoting cell proliferation and differentiation, GFs are known to increase bone healing rate by stimulating the intricate biological cascades that occur during bone regeneration [70]. Many osteogenic growth factors, including bone morphogenetic proteins (BMPs), recombinant human bone morphogenetic proteins (rhBMPs), transforming growth factors (TGFs), insulin-like growth factors (IGFs), and platelet-derived growth factors (PDGF) assist bone injury repair with promoting angiogenesis, osteogenesis, and chondrogenesis by attracting progenitor cells [71,72]. In addition to bone tissue, the osteoconductive BMP subfamily induces bone formation by stimulating pluripotent cells to differentiate into bone-forming cells [73]. In cases of critical size defects, osteocunductive factors are particularly important. In the injured bone, GFs have been incorporated using a variety of approaches [74]. GF-loaded Ca-P coatings have been widely applied to orthopedic and craniofacial implants made from collagen [75]. As one of the transforming BMP superfamily members, rhBMP-2 is the most likely to be studied. It has been reported that rhBMP-2 performed very impressively in simulating the differentiation process of stem cells into bone-forming cells [76]. In contrast to its surface-adsorbed mode, the incorporation of the BMP-2 agents into the octacalcium phosphate coatings structure enhances coralline hydroxyapatite (CHA) granules’ osteoinductivity and biocompatibility [76]. Among metal agents used to combat bacteria, zinc is undoubtedly the most widely used [77]. Studies have found that zinc ions have a longer-lasting bactericidal effect on viable bacteria populations [78,79]. Most recently, through a combination of proteins and ions adhering together by mussel adhesion, as well as a molecular click strategy, an immunomodulatory coating containing immobilized metallic ions (e.g., Zn^2+^) and osteoinductive GFs (e.g., BMP-2 peptide) are designed on the surface of Ti-based bone screw, as seen in Figure 4 [80]. Through macrophage switch from M1 to M2 phenotypes, Zn^2+^ and BMP-2 peptide co-treated implants can promote osteogenic differentiation of bone marrow mesenchymal stem cells (BM-MSCS), improving their mechanical stability in live conditions and improving osseointegration at the interface between bone tissue and implant. In general, the dual-effect coating can provide a novel concept for metallic implants intended for bone tissue engineering applications with osteoinductivity and immunoactivity properties. In order to facilitate osseointegration and bone healing, macrophages regulate the conversion of macrophage phenotypes and create a microenvironment for immune modulation. Recent in vivo studies showed that in rats orally exposed to Zn^2+^ ions for 7 days, cytokines and oxidative stress levels increased, and hepatic and renal tissues showed pathological changes [81].

A dual-layered drug carrier was developed that uses a pore-closed poly(lactic-co-glycolic acid) microparticle-loaded rhBMP-2 (rhBMP-2) filler and a photo-crosslinked CS hydrogel loaded with vancomycin to enhance the antibacterial (*S. aureus*) and osteogenesis performance of dental implants. Bone regeneration is stimulated by BMP-2. Results showed that CS hydrogels containing vancomycin decreased a bacterial number significantly by 88% or 18%, respectively, in comparison to CS hydrogels and PLGA/CS hydrogels containing vancomycin/rhBMP-2. Furthermore, in vitro osteogenic differentiation of MC3T3-E1 cells was demonstrated to be significantly decreased in ALP activity by rhBMP-2-loaded PLGA/CS hydrogel and vancomycin-loaded CS hydrogel, respectively, as compared to CS hydrogel and vancomycin-loaded CS hydrogel, respectively. As a result of the study, Song and Xiao determined that vancomycin-loaded CS hydrogels and vancomycin/rhBMP-2-loaded PLGA/CS hydrogels caused mild inflammation when compared to CS hydrogels, and that the number of inflammatory cells in the vancomycin-loaded CS hydrogel, vancomycin/rhBMP-2-loaded PLGA/CS hydrogels, and CS hydrogels groups were 81.21 ± 6.37%, 14.36 ± 4.53%, and 8.52 ± 2.80%, respectively. The outputs of this study revealed that the double-layered drug carrier released vancomycin rapidly for a period of 2 days and rhBMP-2 for approximately 12 days in a sustained manner, thus exhibiting antibacterial and osteogenic effects. Seeing as how this sequential drug release system may improve the osteointegration of dental implants after surgery, this coating agent for dental implants could potentially be considered to be an attractive coating agent [82].

### 2.4. Anti-Resorptive Drug-Eluting Implants

**Figure 5:**
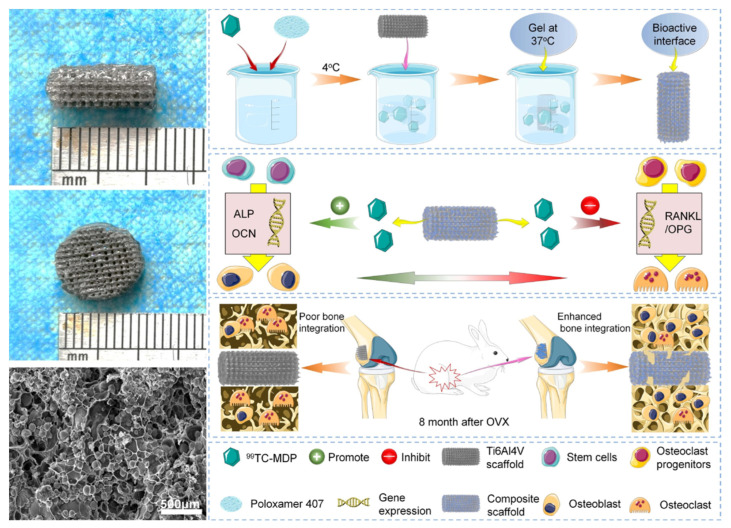
External appearance and representative SEM image of the coated Ti implant, and fabrication of the 99Tc-MDP-loaded hydrogel incorporated bioactive interface (TH/PTI) and its effects of promoting osteogenic differentiation and inhibiting osteoclastogenesis, which results in enhanced osteoporotic bone integration. Reproduce and adapted from [88] under Creative Commons Attribution 4.0 International License (CC BY 4.0).

Bisphosphonates (BPs), usually referred to as antiresorptive drugs, are used in cases of osteoporosis, osteolysis, or hypercalcemia to treat musculoskeletal disorders [81]. BPs can inhibit osteoclast activity, reduce osteoporosis risk, and promote osteogenesis by their structural backbone [83]. BPs are less bioavailable when administered orally or intravenously, which has led to a focus on local delivery as a solution [83,84]. An in vivo study using Ti implants coated with plasma-sprayed HaP revealed increased mechanical fixation and higher peri-implant bone density as a result of BPs added to the HaP coating [85]. Through various signaling pathways, strontium ranelate and simvastatin inhibit bone resorption and promote bone formation [86]. By effectively improving the local bone microenvironment, this implant contains high concentrations of strontium ranelate and simvastatin to enhance osteoporosis patients’ osseointegration [87]. Recently, an inorganic–organic bioactive interface loaded by a newly-developed anti-osteoporosis drug (technetium methylenediphosphonate, 99Tc-MDP) with an anti-osteoporosis property was constructed [88]. The substrate was porous Ti alloy that printed in three dimensions (3D) and loaded with organic temperature-sensitive poloxamer 407 hydrogel, as seen in Figure 5 [88]. Since 3D printing was introduced in the field of biotechnology, it has shown excellent ability in the biomedical engineering and pharmaceutical field because of its high adaptability in utilizing various materials, its ability to develop intricate engineering parts, as well as its high efficiency in terms of time and cost [89,90]. In high concentrations or following burst release of BPs, osteoclasts as well as osteoblasts can undergo apoptosis. The pulse electrodeposition technique allows a more controlled and slower release of zoledronate than the soaking method, so it is ideal for coating and incorporating the drug. In one-step electrochemical deposition of drug coated surfaces, osteoblasts have been shown to proliferate and differentiate osteogenically, but osteoclasts are not significantly inhibited. This may improve bone formation and decrease osteoporosis-related bone resorption near magnesium-based implants [91]. Bioactive interfaces loaded with 99Tc-MDP exhibited the strongest osseointegration with a native bone when implanted into osteoporotic rabbits’ distal femoral defects. In addition, osteoprotegerin /receptor activators were regulated by the drug delivery system to inhibit osteoclastic activities, which significantly reduce the osteoporosis progress rate of the patient and prevented the continuous destruction of bone tissue around the interface through the drug delivery system.

## 3. Most-Studied Drug-Eluting Systems

### 3.1. Titanium-Based Implants

**Figure 6:**
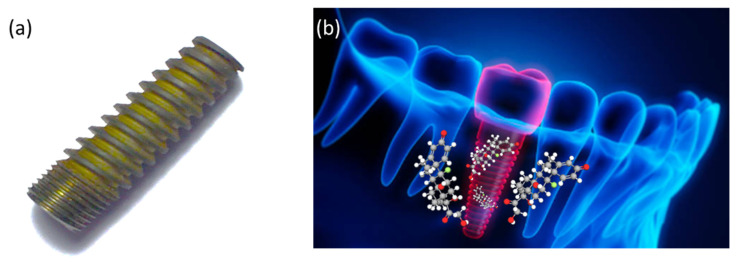
(**a**) DEX-coated Ti dental implant developed by Dr. M. S. Bathaei research group, and (**b**) schematic representation of DEX release in implantation site.

Orthopedic infection prevention is generally achieved through the use of systemic antibiotics (which is the most common) and local antibiotics [92]. There have been recent proposals to coat metallic implant surfaces with controlled antibiotic drug delivery systems [93,94]. A number of advantages are associated with these systems, including controlled release rates and the possibility of coating surfaces with selective agents [95]. It is important to develop antimicrobial surface coatings that maintain or enhance the material biological performance [96]. The application of antimicrobial agents to dental implants may act as a monolithic system since the drug release should be homogenous throughout the whole implant [97,98]. Figure 6a indicates the coated Ti dental implant with dexamethasone (DEX) developed in Dr. M. S. Bathaei group. The drug release of DEX is shown schematically in Figure 6b.

It is also necessary for this system to maintain stable and effective concentration of drug on the site of the implant to prevent the development of bacterial resistance [99,100]. It is important to understand, however, that since dental implants are expected to last for many decades, the drug release coating should be able to recharge/redeposit when needed, otherwise, it will only function during the period of initial healing and the formation of the biofilm atop the implant [101]. Despite the fact that some drug delivery agents have the advantage of enhancing the release of drugs, such as polylactide acid (PDLA), this coating method still suffers from some major disadvantages, including a short-term release and the inability to reload the drug [102]. There has been some promise in treating peri-implant infections with a local drug delivery system comprised of minocycline microspheres, a therapy that has been used for more than 20 years for periodontal disease in teeth [103]. Recently, however, engineering approaches have been developed for coating surfaces with modified materials that are loaded with antibiotics in order to control the formation of biofilms and, consequently, the development of infection associated with implants [104]. It has become increasingly common in recent years to incorporate antibiotics into surface coatings for Ti materials. There have been some difficulties using these coatings because, although they are being evaluated, they are susceptible to short-term release characteristics, resulting in reduced release as well as cytotoxicity because proteins adsorb on top of the coating. A suitable antimicrobial activity must also be determined by taking into account the surface topography properties of these treatments [105]. There seems to be no consensus on the optimal antibiotic and coating technique that should be applied to Ti material to minimize implant-related infections, based on antibiotic and coating technology employed on Ti material. It has also been explored if it is possible to release drugs in advanced ways, including triggered, sequential, and delayed releases [106]. Antibiotic releasing from metallic implants surface have also been shown to possess osseointegration, immunomodulatory, anticancer, and antibacterial properties in numerous in vivo studies [107]. Table 1 summarizes the in vivo studies of various drug coated Ti implants for bone tissue engineering applications. As long as a diffusion gradient exists between the implant surface and the bioactive/therapeutic molecule, any bioactive/therapeutic molecule can theoretically be loaded onto surface of implant for local release in implantation site. A unique characteristic of biopolymers such as CS that has been used as a drug delivery platform on the implant surface is its ability to inhibit bacterial growth, as well as promote osteoblast activity, thus providing dual synergistic benefits: osteogenic and antibacterial [107].

### 3.2. Gentamicin-Eluting Implants

In 1971, Gentamicin (GM) was introduced into parenteral use after being discovered in 1963 [108]. The use of GM in medicine has been widespread since then. Gram-negative bacterial infections are treated with aminoglycosides, the oldest antibiotic. In vitro studies showed that GM induces mesangial cell contraction and reduces filtration [109]. A number of mechanical measures, such as platelet-activating factor, are capable of controlling mesangial contractions, calcium-sensing receptor (CaSR) stimulation, and increased oxygen reactive species (ROS)/oxidative stress [109,110]. As well, a number of studies have demonstrated that calcium channel blockers may inhibit mesangial cell proliferation and contraction when used in conjunction with other therapies. In order to facilitate both of these processes, cells must have an increased level of free calcium (Ca^2+^) in their cytosol [110]. GM increases intracellular Ca^2+^ by releasing internal calcium depots and causing extracellular calcium to enter cells. A rise in calcium levels stimulates the phospholipases, nucleases, and proteases, which disrupt the function of cell membranes and result in more damage to the kidneys during the creation of GM nephrotoxicity [111]. As seen in Table 1, GM is the most used antibiotics on coated implants described in the literature. Some of the metallic implants containing GM-based drug delivery are summarized in Table 2.

**Table 1:** In vivo studies of various drug coated metallic implants for bone tissue engineering applications.

Implant Material	Surface Treatment Method	Antibiotics Drug	Deposition Technology	Vivo Type	N * Number	Surgical Site	Infection Model and System	Follow-Up	Ref.
cpTi	Anodization + alkaly treatment + HA	Tobramycin	Soaking method	Rab	5	F	*S. aureus* (ATCC 6538)	9d	[112]
cpTi	Machined	Vancomycin	Manual application (PH)	Rab	9	R	*S. aureus* (UAMS-1 strain)	1w	[113]
cpTi	PLLA	Rifampicin + Fusidic acid	Solvent-casting	Rab	36	T	*S. aureus* (V 8189-94)	4w	[114]
cpTi	Beadblasted and etched	Vancomycin	Covalent immobilization	Mic	NR	S	*S. aureus* (SH1000) *C. albicans* (SC5314)	2d (fungal) 4d (bacterial)	[115]
cpTi	Machined and nanotubular anodized surface	Gentamicin	Soaking method	Rab	36	T	*S. aureus* (ATCC 25923)	6w	[116]
cpTi	PDLLA	Gentamicin	NR	Rat	30	T	*S. aureus* (ATCC 49230)	6w	[117]
cpTi	Machined or PDLLA	Gentamicin	PDLLA suspension	Rat	30	T	*S. aureus* (ATCC 49230)	6w	[118]
cpTi	Machined + NIR light	Gentamicin	Vacuum drying process onto PEG-MoS_2_ coating + CS	Rat	18	S	*S. aureus* (NR strain origin)	1d, 3d, 1w	[119]
cpTi	Anodized + PLEX	Doxycycline	Spraying	Rab	28 (12 MSSA +16 MRSA)	H	*S. aureus* MSSA (JAR60131) *S. aureus* MRSA (LUH15101)	4w	[120]
Ti6Al4V	Anodized	Vancomycin	Sol-gel	Rat	11	F	*S. aureus* (NR strain origin)	1, 2, 3, 4w	[121]
Ti6Al4V	Machined	Rifampicin + Fosfomycin	Ink-jet	Rab	22 (11 MSSA+ 11 MRSA)	T	*S. aureus* (MSSA EDCC5055) (MRSA T6625930)	4w	[122]
Ti6Al4V	Machined	Vancomycin	Covalently link	Rat	9	F	*S. aureus* (ATCC 25923)	1, 2, 3w	[123]
Ti6Al4V	TiO_2_ nanotubes	Gentamicin + Vancomycin	Drug adsorption	Rab	20	F	*S. aureus* (Human Sa5)	4w	[124]
cpTi	Si-sandblasted	Clindamycin or Teicoplanin	Spraying	Rab	30	T	*S. aureus* (ATCC 29123)	1w	[125]
Ti6Al4V	Porous	Ciprofloxacin	Layered double hydroxides suspension	Mic	12	S	*P. aeruginosa* (PAO1 CTX::lux)	4h	[126]
cpTi	Porous Porous + CS	Vancomycin	Electrophoretic deposition	Rat	18	T	*S. aureus* (ATCC 49230)	4w	[127]
Ti6Al4V	Si-sandblasted	Minocycline + Rifampin	Spraying	Rab	25	F	*S. aureus* (P1—variation of ATCC 25923)	1w	[128]
Ti6Al4V	Plasma chemical oxidation	Gentamicin	Immobilization (TA or SDS)	Rat	15	T	*S. aureus* (ATCC 49230)	4w	[129]
Ti6Al4V	Dopamine methacrylate + PEGDMA-Oligo HYD	Vancomycin	Covalently bond	Mic	22	F	*S. aureus* (Xen 29)	3w	[130]
cpTi	Sandblasted and etched	Gentamicin	Polyelectrolyte adsorption (PEM + PGA/HEP)	Rat	30	T	*S. aureus* (ATCC 49230)	4w	[131]
Ti6Al4V	Plasma-sprayed	Vancomycin	Impregnated on the plasma-sprayed coating	Rab	20	T	*S. aureus* MRSA (ATCC 43300)	6w	[132]
Ti6Al4V	Machined	Vancomycin	Covalently bond	Mic	14	F	*S. aureus* (Xen29)	3w	[133]
Ti6Al4V	PDLLA	Tobramycin	Impregnated on PDLLA coating	Rab	12	T	*S. aureus* (ATCC 25923)	8w	[134]
cpTi	Layer-by-layer	Gentamicin	Polyelectrolyte deposition	Rab	27	F	*S. aureus* (ATCC 49230)	4d, 1w	[135]
Ti6Al4V	Al-blasted + HA	Gentamicin	Spraying + PLGA	Rab	14	F	*S. aureus* (ATCC 25923)	2d, 1w	[136]
Ti6Al4V	Machined	Enoxacin	Covalent immobilization	Rat	24	F	*S. aureus* (ATCC 43300)	3w	[137]
Ti6Al4V	Machined	Bacitracin	Immobilization	Rat	10	F	*S. aureus* (ATCC 25923)	3w	[138]
cpTi	Nanofiber	Doxycycline	Coaxial electrospinning	Rat	48	T	*S. aureus* (ATCC 49230)	4, 8, 16w	[139]
cpTi	PEG-PPS	Vancomycin or Tigecycline	Encapsulation in PEG-PPS solution	Mic	18	F	*S. aureus* (Xen36)	6w	[140]
TiAlNb	Ca-P	Gentamicin	Dip coating	Rat	18	T	*S. aureus* (JAR060131)	1w	[141]
cpTi	Nanotubes	Gentamicin	Lyophilization + Vacuum-drying	Rat	9	F	*S. aureus* (ATCC 25923)	6w	[142]
cpTi	Machined	Vancomycin	Soaking method on nanotubes coating + catechol functionalization	Rat	6	F	*S. aureus* (ATCC 25923)	4w	[143]
cpTi	Machined + NIR light	Daptomycin	Immobilization with IR820 dye on PDA nanocoating	Rat	NR	T	*S. aureus* (ATCC 25923)	2w	[144]

Table notes: Implant surface treatment (TiO_2_, titanium dioxide; Si, Silica; CS, chitosan; PEGDMA, polyethylene glycol dimethacrylate; Oligo, oligonucleotide; HYD, hydrogel; HA, hydroxyapatite; PLLA, poly-L-Lactide; PDLLA, poly(D,L-lactide); NIR, near-infrared light; PLEX, polymer-lipid encapsulation matrix; Al, aluminum; PEG, poly(ethylene glycol); PPS, poly(propylene sulfide); Ca-P, calcium and phosphorus). Deposition technology (TA, tannic acid; SDS, sodium dodecyl sulfate; PEM, polyelectrolyte multilayer; PL, polycation; PGA, polyanion; HEP, heparin; PH, phosphatidylcholine; NR, not reported; PDLLA, poly(D,L-lactide); PEG, polyethylene glycol; MoS2, molybdenum disulfide; CS, chitosan; PLGA, poly(lactic-co-glycolic acid); PPS, poly(propylene sulfide); PDA, polydopamine); Animals (Rat, rats; Mic, mice; Rab, rabbits), Sample number (N) (MRSA, methicillin-resistant *S. aureus*; MSSA, methicillin-sensitive *S. aureus*; NR, not reported), Surgical site (T, tibia; F, femur; S, subcutaneous; H, humerus; R, radius), Follow-up (h, hour; d, day; w, week). ∗ Sample number reported is the total number of infected animals used for microbiological assessments of non-loaded and antibiotic-loaded surfaces.

## 4. Novel Drug-Eluting Implant: Smart Drug Delivery System

**Figure 7:**
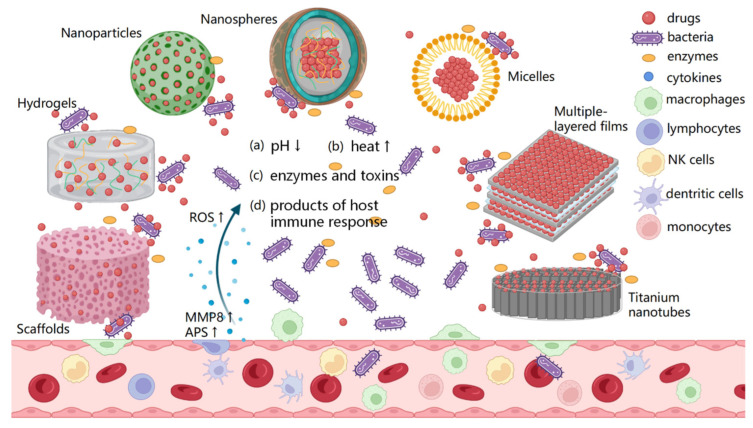
The schematic representation of smart bacteria-responsive drug delivery systems. Scaffolds, hydrogels, nanoparticles, nanosphere, micelles, multiple-layer films and TiO_2_ NT loaded with drugs are triggered by the changes specific to the infection microenvironment, including the (**a**) pH decreasing, (**b**) elevated local temperature, (**c**) bacteria-specific enzymes and toxins and (**d**) products of host immune response, aiming to kill the bacteria. Reproduce and adapted from [150] under Creative Commons Attribution 4.0 International License (CC BY 4.0).

In recent decades, the development of smart metallic implants has become a popular research frontier in biomedical engineering, capable of responding to stimuli and adapting their responses in response to their surroundings. A small external trigger can cause abrupt changes in a smart surface’s properties and alterations in its macroscopic structure as a consequence of its physical properties [148]. Through smart surfaces in drug-eluting implants, the frequency of dosing can be reduced, therapeutic concentrations can be maintained during a single dose, and non-target tissues can be protected from drug accumulation [149]. As a result, smart surfaces are capable of reacting to external stimuli, such as pH, temperature, electric and magnetic fields, light, as well as the concentration of biomolecules, thereby inducing a controlled release of the drug that has been loaded. The schematic illustration of smart drug delivery systems on metallic implants is shown in Figure 7 [150].

A cocktail of enzymes, such as hyaluronidase (HAase) and chymotrypsin, is secreted by pathogens at various stages of colonization and biofilm formation at implant sites. It has been shown that coating implant surfaces in biopolymers or using linkers that can degrade enzymatically with the aid of enzymes can help to facilitate local therapy as soon as an infection occurs. The incorporation of these polymers onto drug-loaded implant surfaces can also enable triggered release since several natural and synthetic polymers can be degraded by enzymes. HA-gen-grafted hyaluronic acid coatings on deferoxamine (DFO)-loaded nanotubes on Ti implants have been reported by Yu et al. [151]. It was found that this structure is able to function as a triggered drug release system in the absence of HAase, but a burst release of DFO was observed in the presence of HAase. A burst release of DFO was enabled by HA-Gen’s degradation in response to infection at the implant surface resulting in the release of gentamicin, which reduced the microbial load and enabled angiogenesis and osteogenesis to rapidly occur.

As a result of bacterial infection, the pH of the local environment may change from a normal physiological value of 7.4 to an acidic value of 5.5. As a result of this shift in pH, local therapy from the implant surfaces has been attempted in several ways. In a recent study by Wang et al., researchers demonstrated that a pH-responsive system can be created by coupling the metal ions Zn^2+^ and Ag^2+^ with a coordination polymer (CP) such as 1,4-bis (imidazol-1-ylmethyl) benzene (BIX) [152]. It was used to load antibacterial nanoparticles and vancomycin into NTs, and these nanotubes were then sealed with antibacterial polymers. Since the coordination bonds are extremely stable at a neutral pH, it is unlikely that much drug will be released. Nevertheless, when acidic conditions are present, the H^+^ ions release the drugs from the NTs by cleaving the coordination bonds. *S. aureus* and *E. coli* antibacterial activity was inversely proportional to acidity and release rates.

Bacterial infections are known to cause an increase in local temperatures, a factor that is also considered to be a trigger for infection. There has been a great deal of interest in smart polymers that undergo phase transitions within a specific range of temperatures when exposed to an abrupt change in temperature. In an aqueous environment, poly(N-isopropylacrylamide) (PNIPAM) can undergo a smooth transition from a two-phase mixture into a one-phase mixture when the temperature decreases below 37 °C. This smart polymer is a good example of such a polymer. The study by Choi et al. found that levofloxacin could be controlled to be released by a brush coating made from poly(di(ethylene glycol) methyl ether methacrylate)) (PDEGMA) [153]. Due to the lower critical solution temperature behavior of the brushes, the localized rise in temperature of the infected site triggers the onset of drug release. In vivo tests with rats infected with *S. aureus* showed levofloxacin had antibacterial activity, and PDEGMA had antifouling effects [154].

## 5. Outlook and Perspectives

Some limitations of the technologies described herein have already been addressed, but many more must be resolved in order to enhance bench-to-bedside progression. By applying micro/nano-technology to encapsulate multi-layered and multi-material templates and through additive manufacturing, advanced encapsulation methods have enabled significant progress towards the delivery of targeted drugs and precise spatiotemporal release control. To adapt to varying implant environments, the drug industry is constantly innovating based on advances in pharmacology and pharmacokinetics. As metallic materials science develops, Ti implant processing technology continues to improve, and a variety of devices that conform to human biomechanics and are capable of storing and slowly releasing drugs have been prepared, resulting in a longer acting time, even up to several months for drug delivery systems and greater stability. A great deal of future research should focus on how Ti implants interact with their drug-loading systems in order to achieve a more holistic approach to the synergy. As such, the implants should be developed that will improve their antibacterial properties, their ability to promote osseointegration, their balance of physical properties, and other tailored requirements, thereby providing comprehensive solutions to the numerous implant properties that are required. Another area of study in drug-eluting implants will be on the adhesion mechanisms of drug molecules on the uncoated and coated metallic materials. Moreover, with predictable release kinetics and more particular therapeutic actions, we may be able to attain more specific therapeutic effects. This proof-of-concept, which incorporates sensing systems to indicate regeneration and healing progresses, predicts the development of multifaceted orthopedic implantable devices that will eventually serve as supplementary functions as well as stimuli-responsive drug delivery for a variety of smart applications. As a result of this future trend, resourceful orthopedic therapies will be fabricated, thereby reducing the social and financial burdens associated with current practices by a significant amount. Providing timely, customized, intelligent treatment, reducing hospitalization time, minimizing cytotoxicity, maximizing long-term implant utility, and reducing post-surgical complications and revision surgeries. In conclusion, the latest developments in pharmacology and metal materials science, combined with the perspective of orthopedics thus far, can aid in the solving of more orthopedic problems in a synergistic manner.

## 6. Conclusions

A synthetic orthopedic and craniofacial implant that will offer impeccable structural support will also be able to assist in the natural healing process by stimulating new bone formation. It can also meditate the body’s response to the wound, reduce the risk of infection, and add additional features based on specific situations as well as obtain faultless structural reinforcement. In bone tissue engineering, finding a solution to integrate multifunctional properties into one platform is crucial to creating smart multifunctional implantable devices that ensure the effective active molecules’ encapsulation and the controlled release of each therapeutic agent at the right time and space. There are several types of metallic drug-eluting implants that are used in orthopedic applications. Generally, the drugs are incorporated into a coating (whether it is either polymeric or ceramic) that is applied onto the metal surface in order to deliver the drug. If bacteria are exposed to suboptimal concentrations for an extended period, they may develop resistance to antibacterial drugs. Therefore, it is imperative not to allow the drug concentration to fall below the therapeutic window. Antibacterial drugs are delivered to implants to prevent bacterial growth and infection, but they should be released within a specific range to provide maximum benefit. Stimuli-responsive or smart drug delivery systems can be substantially expanded with further progress in this field.

## Figures and Tables

**Table 2:** In vivo and in vitro studies of GM-eluting metallic implants for reducing the bacterial activities in implantation site.

Implant Material	Method	Test Model	Bacterial Culture	Outcomes	Ref.
Titanium	GM loaded nanotubes coated over the implant surface	In-vivo	*S. aureus*	Enhanced Bacterial inhibition	[108]
Titanium	GM loaded on the surface via immersion in GM solution	In-vitro	*S. aureus*, *P. aeruginosa* and *S. Epidermidis*	Allowed surface cover with mammalian cells by eradicating bacterial contamination. Use of a combination of Ag-GM to kill the bacteria and eradicate the need for mammalian cells coverage	[109]
Ti6Al4V	GM loaded Fe_3_O_4_/carbonated hydroxyapatite coating	In-vitro	*S. Epidermidis* cell via spread plate method	Resistance to bacterial adhesion and biofilm formation Enhanced biocompatibility and mitogenic activity	[110]
TiO_2_	Porous walls of scaffold impregnated with GM loaded poly (lactide-*co*-glycolide) microparticles	In-vitro	*S. aureus* and *S. Epidermidis* via Agar diffusion test	25% of drug release was during the initial 8 h followed by sustained delivery till 50 days. Practical compatibility with osteoblast cells. Resistance to bacterial activities	[111]
Magnesium foam	Porous Mg scaffold immersion in GM solution	In-vitro	Tested under PBS solution	Pores with 25% porosity can withhold more drugs than those with 10% porosity. The drug holding capacity can be controlled by apparently controlling the content/amount of spacer mixed	[145]
Stainless Steel	Coating of chitosan/gelatin/silica-GM via Electrophoretic deposition	In-vitro	*E. coli* and *S. aureus*	The growth of bacteria was inhibited during and after 30 h of immersion. Cell proliferation was observed during the first 7 days but slowed down after that due to coating reaction or growth starting below the coating. Prevention of bacterial film formation due to 40% release of GM in first 24 h.	[146]
AZ31 Magnesium alloy	Multilayer films of poly (allylamine hydrochloride) (PAH) + poly (acrylic acid) (PAA0 + GM through spin assisted LBL assembly and heat-treated (HT)	In-vitro	*S. Aureus* via plate counting method	The bacterial colonies reduced to 1.3% and 0.5% for (PAH/PAA-GS)10 and HT- (PAH/PAA-GS)10 films, respectively (PAH/PAA-GS)10 demonstrates complete release of drug after 72 h whereas HT- (PAH/PAA-GS)10 have it after 288 h. Decrease in corrosion rates of the alloy	[147]

## Data Availability

Not applicable.
